# Nut Consumption and Sperm Quality in Healthy Men: Results From the Led‐Fertyl Study

**DOI:** 10.1111/andr.70204

**Published:** 2026-02-26

**Authors:** Estefanía Dávila‐Córdova, Nancy Babio, Cristina Valle‐Hita, María Fernández de la Puente, Alfonso Beltran‐Arasa, Miguel Cebrián‐Puig, Violeta Fambuena‐Perez, Iván García‐Serrano, Michelle M. Murphy, Jordi Salas‐Salvadó, Albert Salas‐Huetos

**Affiliations:** ^1^ Departament de Bioquímica i Biotecnologia, Unitat de Nutrició Humana Alimentació, Nutrició, Desenvolupament i Salut Mental ANUT‐DSM Universitat Rovira i Virgili Reus Spain; ^2^ Institut D'investigació Sanitària Pere Virgili (IISPV) Reus Spain; ^3^ Centro de Investigación Biomédica en Red de Fisiopatología de la Obesidad y Nutrición Instituto De Salud Carlos III Madrid Spain; ^4^ Section of Epidemiology Department of Public Health Faculty of Health and Medical Sciences University of Copenhagen Copenhagen Denmark; ^5^ Novo Nordisk Foundation Center for Basic Metabolic Research University of Copenhagen Copenhagen Denmark; ^6^ Departament de Ciències Mèdiques Bàsiques Unitat de Medicina Preventiva i Bioestadística Alimentació, Nutrició, Desenvolupament i Salut Mental ANUT‐DSM Universitat Rovira i Virgili Reus Spain; ^7^ Department of Nutrition Harvard T.H. Chan School of Public Health Harvard University Boston Massachusetts USA

**Keywords:** male fertility, Mediterranean diet, nuts, sperm quality, unsaturated fatty acids

## Abstract

**Background:**

Nuts are rich in antioxidants and other bioactive compounds, and recent evidence suggests that their regular consumption may be associated with sperm quality. However, the current scientific evidence remains limited and inconsistent.

**Objective:**

The study aimed to evaluate the association between nut consumption and sperm quality parameters in healthy men of reproductive age.

**Materials and Methods:**

A cross‐sectional analysis was conducted using the data from 222 young men enrolled in the Led‐Fertyl study. Nut consumption was categorized as < 3, ≥ 3 to < 7, and ≥ 7 servings/week (1 serving = 30 g). The main outcomes were sperm quality parameters (sperm count, concentration, vitality, motility, and normal morphology). Multivariate linear and logistic regression models were fitted to analyze associations.

**Results:**

Total sperm count (*β* = 3.38; 95%CI: 1.59, 5.16) and concentration (*β* = 1.17; 95%CI: 0.15, 2.19) were higher among participants in the highest category of nut consumption (≥7 servings/week) compared to those in the lowest (< 3 servings/week). A similar association was observed when modeling nut consumption as continuous; each additional serving per day was associated with higher total sperm count and concentration (*β *= 2.38; 95%CI: 1.03, 3.72 and *β *= 0.83; 95%CI: 0.06, 1.59, respectively). A theoretical substitution of 1 serving/day of nuts with 1 serving/day of potato chips or pastries was associated with lower total sperm count and concentration. Furthermore, compared to participants in the lowest category of nut consumption, those in the highest were 75% less likely to have abnormal sperm motility (OR: 0.25; 95%CI: 0.07, 0.95) and 69% less likely to have seminogram abnormalities (OR: 0.31; 95%CI: 0.14, 0.68).

**Conclusions:**

Our findings suggest that regular nut consumption is associated with higher total sperm count and concentration in young, healthy men of reproductive age.

## 1 Introduction

Several factors have been associated with male infertility, including genetic factors [1], environmental pollution [2], medical conditions [3], psychological stress [4], and modifiable lifestyle factors such as sedentary lifestyle [5], smoking [6], alcohol consumption, and unhealthy diet [7, 8]. Considered a public health problem worldwide, infertility affects 17.5% of couples of reproductive age [9], and in nearly half of these cases, a male factor is involved [10], often related to the decline of semen quality [11]. Therefore, investigating the relationship between lifestyle and reproductive health is essential.

Among dietary factors, healthy dietary patterns such as the traditional Mediterranean diet (MedDiet) have been extensively studied in relation to fertility and have been associated with improved sperm quality [12]. This dietary pattern is rich in nutrients such as omega‐3 fatty acids and antioxidants (e.g., vitamins C and E, selenium, zinc, polyphenols) that have shown to play a key role in cardiovascular [13] and metabolic health [14].

Importantly, nuts are the only food group that has been shown in randomized controlled trials (RCTs) to improve sperm quality in healthy reproductive‐age men [15, 16], likely through anti‐inflammatory and antioxidant mechanisms [17].

However, to the best of our knowledge, no observational studies have specifically evaluated the association between nut consumption and sperm quality or reproductive outcomes in healthy men from the general population who were not selected based on their fertility status. The existing evidence is limited and inconsistent, and comes largely from studies conducted in cohorts of infertile men [18, 19], whose findings may not be generalizable to the wider population. Considering this gap in the literature, the aim of our study is to examine the association between nut consumption and sperm quality among healthy men of reproductive age from the general population. We hypothesized that a high versus low nut consumption would be associated with better sperm quality parameters.

## 2 Material and Methods

### 2.1 Study Design and Participants

The Led‐Fertyl (Lifestyle and environmental determinants of seminogram and other male fertility‐related parameters) study is a cross‐sectional cohort study designed to identify and quantify dietary and other lifestyle factors associated with semen quality parameters. In the present study, we included a total of 222 participants who were enrolled in the Led‐Fertyl study between February 2021 and December 2024 (semen samples and dietary assessments were collected concurrently). These participants were healthy male volunteers of reproductive age between 18 and 40 years. Participants were excluded if any of the following criteria applied: (1) illiteracy or inability/unwillingness to provide written informed consent; (2) institutionalization (defined as permanent or long‐term residence in a care facility); (3) any type of disease in the reproductive history or vasectomy; (4) a documented history of prior cardiovascular disease, symptomatic peripheral arterial disease requiring surgery or diagnosed by vascular imaging, ventricular arrhythmia, uncontrolled atrial fibrillation, congestive heart failure, hypertrophic cardiomyopathy, or a history of aortic aneurysm measuring ≥5.5 cm in diameter or aortic aneurysm surgery; (5) active malignant neoplasm; (6) inability to attend scheduled visits; (7) history of small or large bowel resection or inflammatory bowel disease; (8) immunodeficiency or HIV‐positive status, cirrhosis or liver failure; (9) severe psychiatric disorders; (10) any serious comorbidity with a life expectancy of less than 24 months; (11) history of major organ transplantation; (12) concurrent treatment with immunosuppressants, cytotoxic drugs, etc.; (13) participants with an acute infection or inflammation; or (14) any other condition that could interfere with the study protocol. All participants provided informed consent, both online and in writing.

The protocol adheres to the principles of the Declaration of Helsinki and was approved by the Ethics Committee of the Institut d'Investigació Sanitària Pere i Virgili (CEIm‐IISPV, Ref. 181/2019).

### 2.2 Exposure: Nut Consumption

A validated semi‐quantitative 143‐item food frequency questionnaire (FFQ) [20] was administered by trained dietitians through individual interviews to assess habitual dietary consumption over the previous year. Total nut consumption was calculated by summing the intake of almonds, pistachios, walnuts, and other nuts (e.g., hazelnuts, cashews, Brazil nuts, and peanuts, among others). For each type of nut, consumption frequency was recorded using nine predefined categories ranging from “never or almost never” to “≥6 servings/day,” without distinguishing between methods of preparation (e.g., raw, roasted, fried, or salted). One standard serving was considered equivalent to 30 g.

Nut intake data were converted to grams per day by multiplying the consumption frequency by the standard portion size of each item. Considering the globally recommended intake levels and prior literature [21, 22], total nut consumption was categorized according to consumption frequency into three groups: < 3, ≥ 3 to < 7, and ≥ 7 servings/week. This categorization approach improves the interpretability of the results. Participants who reported nut consumption greater than or equal to 100 g/day were excluded from the primary analysis. This threshold was applied to exclude biologically implausible dietary reports and potential outliers, as such extreme consumption levels are unlikely to reflect the habitual dietary intake of this population.

### 2.3 Outcome: Sperm Quality Parameters

The main outcomes of this study were total sperm count, concentration, motility, vitality, and normal morphology.

Conventional semen parameters were assessed according to the fifth edition of the *WHO Laboratory Manual for the Examination and Processing of Human Semen* [23]. One complete semen sample was collected from each participant, via masturbation into a sterile plastic container in a designated private room near the laboratory, after a period of sexual abstinence of 3–7 days. Analyses were performed within 60 min after collection using fresh samples. Only one complete semen sample per participant was included in the study.

Sperm concentration, motility, and morphology were evaluated using the Computer‐Aided Sperm Analysis (CASA) SCA system (version 6.5.0.67, Microptic, Barcelona, Spain), in combination with an Olympus CX43 phase‐contrast microscope (EVIDENT Corporation, Tokyo, Japan). Total sperm count (millions of spermatozoa per ejaculate [×10^6^ spz/ej]) was calculated by multiplying the ejaculate volume (mL) by the sperm concentration (×10^6^ spz/mL). Sperm motility was graded as progressive motility, non‐progressive motility, or immotile spermatozoa, and was expressed as a percentage. Total motility was calculated as the sum of progressive and non‐progressive motility. Sperm vitality was determined using the hypoosmotic swelling (HOS) test, and the results were expressed as a percentage of viable spermatozoa. Sperm morphology was evaluated using the Hemacolor (Merck KGaA, Darmstadt, Germany) staining protocol. Spermatozoa were classified as normal or abnormal based on the presence of defects in the head, midpiece, or tail (single or combined). Sperm morphology was expressed as the percentage of spermatozoa with normal morphology. The motility, vitality, and morphology parameters were evaluated in, at least, 200 spermatozoa.

Sperm quality parameters were also classified based on threshold values defined by the fifth edition WHO 2010 [23] reference values (fifth percentile): sperm concentration < 15 × 10^6^ spz/mL, sperm count < 39 × 10^6^ spz/ejaculate, sperm vitality < 58%, total motility < 40%, progressive motility < 32%, and normal morphology < 4%. The seminogram was classified as abnormal if any of the parameters (total sperm count, concentration, vitality, motility, or morphology) fell outside the reference limits described above. Using these reference values, we created dichotomous variables (abnormal/normal) for further analysis.

### 2.4 General Characteristics and Covariate Assessment

Body composition (weight, height, and waist circumference) and blood pressure were measured using standardized methods by trained dietitians during a face‐to‐face visit. Body mass index (BMI) was calculated by dividing weight in kilograms by height in meters squared (weight [kg]/height [m^2^]) and categorized according to WHO criteria as follows: underweight (<18.5 kg/m^2^), normal weight (18.5–24.9 kg/m^2^), overweight (≥25.0 to <30.0 kg/m^2^), and obesity (≥30.0 kg/m^2^). Blood pressure was measured twice, in the seated participant, at 5‐min intervals using a semi‐automatic sphygmomanometer. In line with the Physical Activity Guidelines, the physical activity goal was offset at 500 metabolic equivalent (MET) minutes per week and was measured by the validated Regicor Short Physical Activity Questionnaire [24]. Adherence to the MedDiet was assessed through interviews conducted by trained dietitians using the validated Mediterranean Diet Adherence Screener (MEDAS) [25].

### 2.5 Statistical Analysis

Data analysis was performed using the Led‐Fertyl database, which was updated in December 2024. The sample size for the present study was calculated to detect significant differences in sperm progressive motility based on the results reported by [26]. Considering the variability observed in previous large‐scale semen analyses [27] and applying a margin of error of 7% at a 95% confidence level, a minimum of 192 participants was estimated to provide sufficient statistical power (> 80%). With the actual enrollment of 222 participants, the study achieves a more precise estimate of sperm progressive motility within the target population. For missing values, smoking status was imputed by assigning the most frequent category (non‐smokers).

Categorical variables were presented as a number (percentage), while continuous variables were presented as means (standard deviations) or medians [25th–75th percentiles], depending on whether they were normally distributed. The normality of the distributions was evaluated using the Kolmogorov–Smirnov test. Non‐normally distributed outcome variables were cubic root transformed to approximate a Gaussian distribution. The baseline characteristics of the study population were compared between categories of nut consumption using one‐way anova, Kruskal–Wallis test, or chi‐squared, as appropriate. Statistical analyses were performed using IBM‐SPSS (version 27.0, SPSS Inc., Chicago, IL, USA) and STATA (version 14.0, StataCorp LLC, Texas, USA). All tests were considered statistically significant if the two‐tailed p‐value was < 0.05.

#### 2.5.1 Primary Analyses

Multivariate linear regression models were fitted to analyze the associations between categories of nut consumption and sperm quality parameters. The results were expressed as *β*‐coefficients and their 95% confidence intervals (CIs). The linear trend (*p*‐trend) was then calculated by assigning median values to each nut consumption category, treating these values as a continuous variable across the groups within the linear regression models. In addition, categories of nut consumption and nut subtypes (almonds, pistachios, walnuts, and other nuts) were modeled as a continuous variable for each 1 serving/day increase, and the results were also expressed using *β*‐coefficients and 95% CIs. The above linear regression analyses used root‐transformed sperm count and concentration, vitality, and normal sperm morphology. Multivariate logistic regression models were fitted to estimate odds ratio (OR) and their 95% CIs for the associations between categories of nut consumption and dichotomized sperm quality parameters defined according to the WHO 2010 reference thresholds [23]. All regression models were adjusted for several potential confounders. Model 1 was adjusted for age (years), smoking status (current, former, never), education (high school or less, college or higher education), BMI (kg/m^2^), physical activity (METs min/week), and sexual abstinence (days). Model 2 was additionally adjusted for energy intake (kcal/day) and dietary factors such as consumption of vegetables, fruits, legumes, cereals, oils and fats, biscuits, dairy, meat, fish, and beverages.

#### 2.5.2 Exploratory Analyses

A stratified analysis was performed to assess potential associations, categorizing participants into high adherence (≥7 points) and low adherence (≤7 points) groups using the median of the MEDAS score (7 points) as the cutoff and excluding the item related to nut consumption. The models were adjusted for the aforementioned confounding factors. Finally, a theoretical mathematical substitution model was applied to estimate the association between replacing one daily serving of nuts with one daily serving of a commonly consumed snack food, such as potato chips, pastries, or yogurt, and sperm quality parameters. Specifically, the consumption variables in grams per day were transformed into standardized servings per day using the following equivalents: 30 g for nuts, 50 g for potato chips and processed pastries, and 125 g for yogurt. Pastries included croissants, tea pastries, and industrial baked goods, and yogurt included whole and skimmed yogurt. The theoretical substitution of one food group with another was evaluated by including both food groups as continuous variables in the regression model simultaneously. The theoretical substitution effect for each substitution was estimated using the difference between their *β*‐coefficients from the main model, along with the corresponding variances and covariances, to calculate the resulting *β*‐coefficient and its 95% CI.

## 3 Results

Of the total 226 participants, three were removed from the analysis: two with azoospermia and one with no vitality data. In addition, one participant reporting nut consumption ≥ 100 g/day was excluded (210 g/day, representing > 35% of total energy intake). Data from 222 participants were included in the analysis. The flow chart showing participant selection is presented in Figure S1.

Table 1 shows the general characteristics of the study population. The mean (±SD) age was 28.2 ± 5.5 years, and the mean (±SD) BMI was 24.6 ± 3.3 kg/m^2^, 43.2% of the participants were individuals with overweight or obesity. Most of the participants were single (86.9%), non‐smokers (69.8%), and had a university degree (63.1%). In addition, 39.6% had at least one major seminogram parameter (volume, sperm count, concentration, vitality, total motility, progressive motility, or normal morphology) below the WHO 2010 reference values. Table 1 also reports the general characteristics of participants according to the category of nut consumption. Participants in the highest category of nut consumption were more likely to have a higher total sperm count and energy intake (kcal/day). The median [25th, 75th percentile] nut consumption was 17 [6, 30] g/day; approximately half a serving per day (0.57 serving/day).

**TABLE 1:** Demographic, reproductive, and dietary characteristics of the Led‐Fertyl population according to categories of total nut consumption.

		Categories of total nut consumption (servings/week)^a^			
General characteristics	All population (*n* = 222)	<3 servings/week (*n* = 88)	≥ 3 to <7 servings/week (*n* = 60)	≥7 servings/week (*n* = 74)	*p* value^b^
Age (years)	28.2 ± 5.5	28.1 ± 5.7	27.9 ± 5.1	28.5 ± 5.8	0.132
BMI (kg/m^2^)	24.6 ± 3.3	24.6 ± 3.4	24.7 ± 3.3	24.5 ± 3.2	0.964
Waist circumference (cm)	83.6 ± 8.4	83.8 ± 8.3	83.4 ± 8.7	83.3 ± 8.2	0.913
Systolic blood pressure (mmHg)	127 ± 10	127 ± 10	128 ± 11	127 ± 11	0.800
Diastolic blood pressure (mmHg)	74 ± 9	74 ± 8	75 ± 10	72 ± 10	0.122
Physical activity (METs min/week)	3750 ± 2954	3534 ± 3116	3582 ± 2667	4144 ± 2975	0.373
Sleep (h/day)	7.4 ± 0.8	7.4 ± 0.7	7.5 ± 0.8	7.3 ± 0.8	0.624
Weight status					
Normal weight/thinness	126 (56.8)	47 (53.4)	35 (58.3)	44 (59.5)	0.711
Overweight/obesity	96 (43.2)	41 (46.6)	25 (41.7)	30 (40.5)	
Smoking status					
Never smoker	155 (69.8)	57 (64.8)	39 (65.0)	59 (79.7)	0.207
Current smoker	26 (11.7)	15 (17.1)	6 (10.0)	5 (6.8)	
Former smoker	30 (13.5)	11 (12.5)	12 (20.0)	7 (9.5)	
No reported	11 (5.0)	5 (5.7)	3 (5.0)	3 (4.1)	
Education					
High school or less	82 (36.9)	37 (42.1)	20 (33.3)	25 (33.8)	0.441
College or third‐level education	140 (63.1)	51 (58.0)	40 (66.7)	49 (66.2)	
Civil status					
Single	193 (86.9)	80 (90.9)	47 (78.3)	66 (89.2)	0.224
Married	26 (11.7)	7 (8.0)	12 (20.0)	7 (9.5)	
Other	3 (1.4)	1 (1.1)	1 (1.7)	1 (1.4)	
Monthly Income					
Less than €1000	32 (14.4)	11 (12.5)	8 (13.3)	13 (17.6)	0.217
Between €1000 and €2000	142 (64.0)	64 (72.7)	36 (60.0)	42 (56.8)	
More than €2000	48 (21.6)	13 (14.8)	16 (26.7)	19 (25.7)	
Seminogram parameters					
Sexual abstinence (days)	4 [3, 5]	4 [3, 5]	4 [3, 4]	4 [3, 5]	0.611
pH	8.5 [8, 8.5]	8.5 [8, 8.5]	8.5 [8.5, 8.5]	8.5 [8, 8.5]	0.156
Volume (mL)	3.5 [2.5, 4.5]	3.3 [2.4, 4.4]	3.5 [2.3, 4.8]	3.5 [2.9, 4.7]	0.254
Volume < 1.5 mL	6 (2.7)	2 (2.3)	3 (5.0)	1 (1.4)	0.411
Total sperm count (×10^6^ spz.)	159.4 [98.0, 272.5]	135.5 [81.7, 221.8]	146.5 [86.9, 290.6]	210.3 [114, 312.5]	**0.006 **
Total sperm count < 39 × 10^6^ spz.	17 (7.7)	8 (9.1)	7 (11.7)	2 (2.7)	0.123
Sperm concentration (×10^6^ spz./mL)	47.9 [29, 81.8]	42.7 [28.7, 69.3]	50.1 [25.9, 90.1]	55.9 [31.4, 85.2]	0.167
Sperm concentration < 15 × 10^6^ spz./mL	20 (9.0)	9 (10.2)	8 (13.3)	3 (4.1)	0.154
Vitality (%)	83 [76, 90]	82.5 [73.8, 87.8]	85 [76, 91]	82.8 [79, 89.5]	0.618
Vitality < 58%	9 (4.1)	4 (4.6)	3 (5.1)	2 (2.7)	0.756
Total motility (%)	59.9 ± 16.7	58.4 ± 16.5	60.7 ± 18.6	60.9 ± 15.3	0.588
Total motility < 40% motile	26 (11.7)	12 (13.6)	9 (15.0)	5 (6.8)	0.259
Progressive motility (%)	43.3 ± 16.8	42.5 ± 16.1	44.2 ± 18.7	43.4 ± 16.0	0.829
Progressive motility < 32% motile	61 (27.5)	25 (28.4)	17 (28.3)	19 (25.7)	0.914
Non‐progressive motility (%)	16.9 ± 6.6	16.3 ± 6.0	17.1 ± 6.9	17.4 ± 7.2	0.563
Normal sperm morphology (%)	8.5 [5, 15]	7.8 [4.5, 13.5]	10.3 [6, 15.5]	8 [5, 16.5]	0.227
Normal sperm morphology < 4%	27 (12.2)	14 (15.9)	4 (6.7)	9 (12.3)	0.241
Seminogram abnormality	88 (39.6)	41 (46.6)	25 (41.7)	22 (29.7)	0.086
Dietary parameters					
Energy intake (kcal/day)	2592 ± 628	2492 ± 556	2626 ± 662	2825 ± 651	**0.008 **
Total nuts (g/day)	17 [6, 30]	6 [4, 8]	17 [15, 21]	39 [30, 47]	**< 0.001**
Almonds	2 [0, 4]	2 [0, 2]	4 [0, 4]	13 [4, 13]	**< 0.001**
Pistachios	0 [0, 2]	0 [0, 2]	2 [0, 4]	2 [0, 4]	**< 0.001**
Walnuts	4 [0, 13]	2 [0, 2]	4 [0, 13]	13 [13, 13]	**< 0.001**
Others	4 [2, 13]	2 [0, 2]	4 [4, 13]	13 [13, 13]	**< 0.001**
MEDAS score (14 points)	8 [7, 9]	7 [6, 8]	8 [7, 9]	9 [8, 10]	**< 0.001**
MEDAS score (13 points without nut item)	7 [6, 9]	7 [6, 8]	7 [6, 8]	8 [7, 9]	**0.002**

*Note*: Continuous variables are presented as means ± SD or medians [25th–75th percentiles], and categorical variables are presented as number (*n*) and percentages (%). Bolded values indicate *p* < 0.05.

Abbreviations: BMI, body mass index; MEDAS, Mediterranean diet adherence screener; METs min, metabolic equivalent of task minutes; *n*, number of subjects; SD, standard deviation; spz, spermatozoa.

^a^ 1 serving = 30 g.

^b^ *p* value for the differences between categories of total nut consumption was calculated by Pearson's chi‐squared test for categorical variables and a one‐way analysis of variance (anova) or Kruskal–Wallis test for continuous variables with normal or non‐normal distributions, as appropriate.

With regard to the primary analysis, Table 2 reports the associations (*β*‐coefficients; 95% CIs) between categories of total nut consumption (servings per week) and sperm quality parameters. The fully adjusted model showed higher total sperm count (*β* = 3.38; 95% CI: 1.59, 5.16), and sperm concentration (*β* = 1.17; 95% CI: 0.15, 2.19) among participants in the highest category of the nut consumption category, compared to those in the lowest category. Similarly, in the continuous analysis, each 1 serving/day increase in nut consumption was positively associated with total sperm count (*β *= 2.38; 95% CI: 1.03, 3.72) and sperm concentration (*β* = 0.83; 95% CI: 0.06, 1.59) (Table 2).

**TABLE 2:** Associations (*β*‐coefficients and 95% confidence intervals) between nut consumption (categorized by servings per week and modeled continuously per 1 serving/day increase) and sperm quality parameters.

		Categories of total nut consumption (servings/week)				Continuous (1 serving/day)
Sperm quality parameters		<3 servings/week (*n *= 88)	≥3 to <7 servings/week (*n* = 60)	≥7 servings/week (*n* = 74)	*p* trend	
Total sperm count (×10^6^ spz.)^a^	Crude model	Ref.	1.19 [–0.58, 2.95]	2.91 [1.25, 4.58]	**0.001**	**1.87 [0.72, 3.01]**
	Model 1	Ref.	1.23 [–0.49, 2.95]	3.04 [1.40, 4.67]	**< 0.001**	**1.87 [0.73, 3.02]**
	Model 2	Ref.	1.55 [–0.19, 3.29]	3.38 [1.59, 5.16]	**< 0.001**	**2.38 [1.03, 3.72]**
Sperm concentration (×10^6^ spz./mL)^a^	Crude model	Ref.	0.54 [–0.43, 1.51]	0.94 [0.03, 1.85]	**0.042**	0.59 [–0.04, 1.21]
	Model 1	Ref.	0.56 [–0.41, 1.53]	0.98 [0.06, 1.90]	**0.036**	0.57 [–0.07, 1.22]
	Model 2	Ref.	0.70 [–0.29, 1.69]	1.17 [0.15, 2.19]	**0.022**	**0.83 [0.06, 1.59]**
Sperm vitality (%)^a^	Crude model	Ref.	0.08 [–0.16, 0.31]	0.09 [–0.13, 0.30]	0.425	0.01 [–0.14, 0.16]
	Model 1	Ref.	0.05 [–0.19, 0.28]	0.06 [–0.17, 0.28]	0.614	−0.01 [–0.16, 0.15]
	Model 2	Ref.	0.04 [–0.20, 0.28]	0.03 [–0.21, 0.27]	0.785	−0.06 [–0.24, 0.12]
Total motility (%)	Crude model	Ref.	2.24 [–3.27, 7.75]	2.46 [–2.73, 7.65]	0.341	−0.31 [–3.87, 3.26]
	Model 1	Ref.	2.92 [–2.58, 8.43]	3.36 [–1.86, 8.59]	0.196	0.54 [–3.11, 4.20]
	Model 2	Ref.	3.16 [–2.46, 8.78]	4.82 [–0.94, 10.58]	0.095	1.54 [–2.80, 5.89]
Progressive motility (%)	Crude model	Ref.	1.70 [–3.85, 7.25]	0.94 [–4.29, 6.17]	0.706	−1.21 [–4.79, 2.37]
	Model 1	Ref.	2.15 [–3.47, 7.76]	1.67 [–3.66, 7.00]	0.520	−0.61 [–4.33, 3.11]
	Model 2	Ref.	2.75 [–3.01, 8.50]	3.59 [–2.31, 9.50]	0.219	0.77 [–0.368, 5.21]
Non‐progressive motility (%)	Crude model	Ref.	0.80 [–1.39, 2.99]	1.08 [–0.99, 3.14]	0.297	0.59 [–0.83, 2.00]
	Model 1	Ref.	0.98 [–1.22, 3.18]	1.26 [–0.83, 3.35]	0.228	0.86 [–0.60, 2.31]
	Model 2	Ref.	0.53 [–1.70, 2.76]	0.67 [–1.62, 2.96]	0.552	0.25 [–1.47, 1.97]
Normal sperm morphology (%)^a^	Crude model	Ref.	0.29 [–0.07, 0.65]	0.16 [–0.18, 0.51]	0.319	−0.01 [–0.24, 0.23]
	Model 1	Ref.	0.26 [–0.11, 0.62]	0.16 [–0.19, 0.51]	0.330	−0.01 [–0.25, 0.23]
	Model 2	Ref.	0.25 [–0.12, 0.61]	0.17 [–0.21, 0.54]	0.344	0.01 [–0.28, 0.29]

*Note: β*‐Coefficients were estimated using multivariate linear regression models. The linear trend (*p* trend) was calculated by assigning median values to each nut consumption category and treating these values as a continuous variable across the groups in the linear regression models. Model 1 was adjusted for age (years), smoking status (current, former, never), education (high school or less, college or higher education), BMI (kg/m^2^), physical activity (METs min/week), and sexual abstinence (days). Model 2 was additionally adjusted for energy intake (kcal/day) and dietary factors (consumption of vegetables, fruits, legumes, cereals, oils and fats, biscuits, dairy, meat, fish, and beverages). Bold values indicate *p* < 0.05.

Abbreviations: BMI, body mass index; *n*, number of subjects; Ref, reference; spz, spermatozoa.

^a^ Total sperm count, concentration, vitality, and normal morphology were root‐square transformed to approximate a normal distribution.

Table S1 presents sub‐analyses assessing the association between each type of nut and sperm quality parameters, modeled continuously per 1 serving increase per day. Positive associations with total sperm count for almond consumption (*β* = 4.36; 95% CI: 0.66, 8.07), pistachio consumption (*β* = 6.97; 95% CI: 1.83, 12.12), and consumption of other nuts (*β* = 3.30; 95% CI: 0.48, 6.11) were found. No significant associations were found between walnuts consumption and sperm quality parameters.

Moreover, participants in the highest total nut consumption category were 75% less likely to have abnormal total motility (OR: 0.25; 95% CI: 0.07, 0.95). The association was also significant for a seminogram classified as abnormal (OR: 0.31; 95% CI: 0.14, 0.68) (Figure S2).

In participants with a high adherence to the MEDAS score (≥ 7 points), a positive association between higher nut consumption and total sperm count (*β* = 3.67; 95% CI: 1.53, 5.82) and sperm concentration (*β* = 1.67; 95% CI: 0.44, 2.90) was shown, compared to those in the lowest category. Similarly, in the continuous analysis, each 1 serving/day increase in nut consumption was positively associated with total sperm count (*β* = 0.17; 95% CI: 0.06, 0.28) and sperm concentration (*β* = 0.28; 95% CI: 0.01, 0.14), among participants with high MEDAS adherence (Table S2).

**FIGURE 1:**
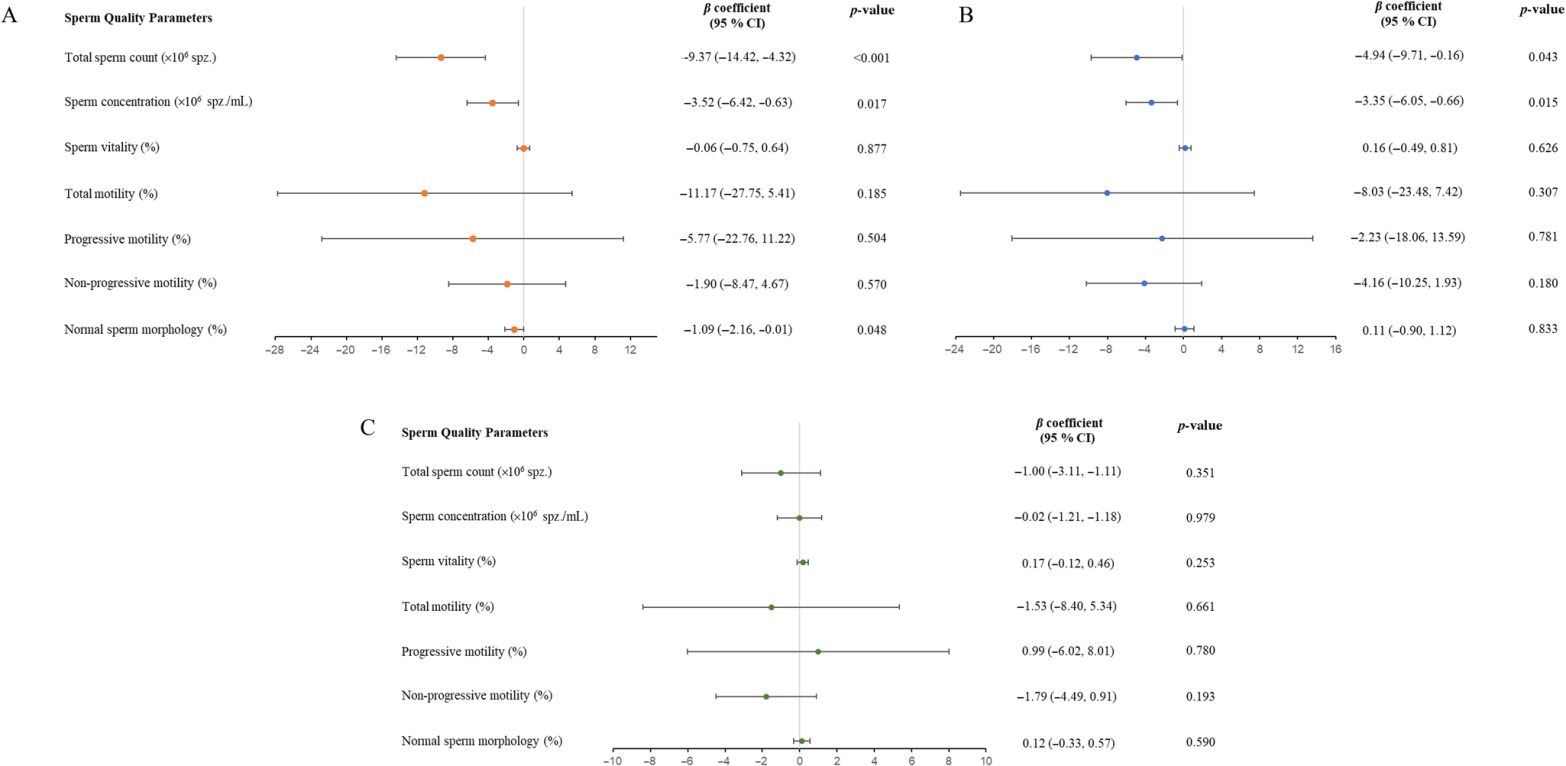
*β*‐Coefficients and 95% CI for sperm quality parameters when substituting one serving/day of nuts with one serving/day of potato chips (A), pastries (B), or yogurt (C). Model was adjusted for age (years), smoking status (current, former, never), education (high school or less, college or high education), BMI (kg/m^2^), physical activity (METs min/week), sexual abstinence (days), energy intake (kcal/day), and dietary factors (consumption of vegetables, fruits, legumes, cereals, oils and fats, biscuits, dairy, meat, fish, and beverages). BMI, body mass index; METs, metabolic equivalents; spz, spermatozoa.

Finally, in the exploratory analysis, theoretical substitution models adjusted for potential confounders showed that replacing one daily serving of nuts with one daily serving of potato chips was associated with lower total sperm count (*β* = −9.37; 95% CI: −14.42, −4.32), lower sperm concentration (*β* = −3.52; 95% CI: −6.42, −0.63), and lower normal sperm morphology (β = −1.09; 95% CI: −2.16, −0.01) (Figure 1A). Similarly, negative associations were observed when nuts were substituted with pastries (lower total sperm count [*β* = −4.94; 95% CI: −9.71, −0.16] and lower sperm concentration [*β* = −3.35; 95% CI: −6.05, −0.66]) (Figure 1B). However, no significant associations were found when substituting nuts with yogurt for any sperm quality parameters, although a similar trend to potato chips and pastries was observed (Figure 1C).

## 4 Discussion

In this cross‐sectional study, we found that higher nut consumption (≥ 7 servings/week) was positively associated with total sperm count and concentration, compared to lower nut consumption (< 3 servings/week), even after adjusting for sociodemographic, lifestyle, and dietary factors. These findings remained consistent when the categories of nut consumption and each type of nut (almonds, pistachios, and other nuts) were analyzed continuously, reinforcing a linear trend. In addition, theoretical substitution of one daily serving of nuts with potato chips or pastries revealed significant negative associations with sperm quality parameters. Therefore, our results support the hypothesis that regular nut consumption may be associated with better sperm quality parameters.

Our findings are consistent with previous literature, particularly with the FERTINUTS study [16] results, a RCT in which supplementing 60 g/day of mixed nuts for 14 weeks was found to significantly improve sperm quality parameters in healthy men following a Western diet. This is supported by a recent meta‐analysis [28], including the FERTINUTS study and another RCT [15], suggesting the potential positive effects of nut consumption on sperm quality.

However, in two observational studies conducted in infertile men, no significant associations between nut consumption and sperm quality were reported [18, 19]. These discrepancies may be explained by differences in the populations studied. The RCTs included healthy reproductive‐aged men without underlying pathologies, whereas the observational studies were conducted in participants attending infertility clinics. Furthermore, unlike the RCTs, which supplemented with 60–75 g/day of nuts under controlled conditions, our study examined habitual nut consumption as part of a general diet. Our results suggest that a regular intake of nuts (≥ 7 servings per week, or approximately 30 g/day) may be sufficient to induce beneficial effects on sperm parameters in healthy men. This aligns with the general international recommendation for nut consumption (≥ 30 g/day) [29].

In addition, our results are supported by a recent preconception cohort study [30], in which it was reported that consumption of ≥ 25 g/day of nuts and seeds was associated with increased fecundability in men from the general population. Notably, this association persisted independently of omega‐3 polyunsaturated fatty acids intake, suggesting that other bioactive compounds present in nuts, such as antioxidants (vitamin E, vitamin C, selenium, zinc, and polyphenols), dietary fiber, and amino acids like L‐arginine, could play a crucial role in supporting male reproductive function. This could also help explain the results observed in our continuous analysis of each type of nut. We found positive associations between the consumption of almonds, pistachios, and other nuts and total sperm count, while we did not observe a significant association with walnut consumption, despite the fact that a previous RCT had reported a beneficial effect of walnuts on semen quality parameters [15]. One possible explanation is that, although walnuts are particularly rich in omega‐3 fatty acids, almonds and pistachios provide higher amounts of vitamin E, fiber, minerals (such as magnesium and zinc), and polyphenols.

This biological mechanism is supported by experimental and clinical studies showing that nuts can reduce oxidative stress, which is recognized as one of the key contributors to male infertility [14, 16, 28]. For example, the antioxidants found in nuts may protect spermatozoa cells from reactive oxygen species (ROS), thereby preserving sperm DNA integrity and sperm function [31, 32]. Moreover, l‐arginine, a nitric oxide precursor, has been associated with improved testicular blood flow and function, while zinc can help regulate sperm DNA stability [33].

In line with these mechanisms, our theoretical substitution model showed that replacing one daily serving of nuts (30 g) for one daily serving of potato chips (50 g) or pastries (50 g), two typical Spanish unhealthy snacks, was negatively associated with total sperm count and concentration. This suggests that consuming a handful of nuts instead of other unhealthy snacks may be positively associated with sperm quality. Indeed, previous studies have shown that the consumption of ultra‐processed foods is associated with lower sperm quality [26, 34, 35]. However, the theoretical substitution of nuts (30 g) with yogurt (125 g) showed no statistically significant association with sperm quality parameters. This may be because the nutrient profile of yogurt is more favorable than that of the other snack foods evaluated [36]. Yogurt provides high‐quality protein, calcium, and probiotics, nutrients that may support reproductive health, thereby counterbalancing the potential benefits of nut substitution and reducing the observed effect [37]. Consistent with this, previous studies examining yogurt consumption and semen quality have reported inconsistent results [38, 39]. Such discrepancies may reflect differences in the nutritional composition of dairy products, as well as variations in the populations studied.

The sensitivity analysis, which found that the positive association between nut consumption and sperm parameters was evident only among men with high adherence to the MedDiet, refines the primary analysis. This pattern suggests that nuts do not merely act as a marker of an overall healthy diet, but may also have a synergistic or threshold association, whereby their benefits are most noticeable when nuts are consumed as part of a high‐quality dietary pattern. Collectively, these results emphasize the importance of protecting male reproductive function by incorporating healthy foods such as nuts and limiting ultra‐processed products. This is also consistent with our previous research into the relationship between a Mediterranean dietary pattern characterized by adequate nut intake and minimal processed foods, and better sperm quality parameters [8].

The present study has several limitations that should be acknowledged. First, its cross‐sectional design precludes the establishment of causal relationships. Second, dietary intake was assessed using a single FFQ. Although this FFQ has been validated and administered by trained dietitians, it relies on memory and self‐reporting, which makes it susceptible to measurement errors, misclassification, and recall bias. These limitations may have attenuated the observed associations. To minimize the impact of extreme reporting errors inherent in FFQ data, one participant who reported a biologically implausible nut intake of ≥ 100 g/day was excluded from the primary analysis; also, the FFQ did not distinguish how the nuts were prepared (raw, roasted, or fried). This lack of detail may have attenuated the observed associations and should be considered when interpreting the results. Third, our study is based on the analysis of a single semen sample per participant. Although standardized protocols were followed, including monitoring abstinence time, we acknowledge the well‐known intra‐individual variability in semen parameters. Therefore, interpretation of effect sizes must consider this limitation. Fourth, although in our study we have observed associations between nut consumption and some sperm parameters after adjusting for several confounders, we cannot rule out the possibility of residual confounding, meaning that some of the observed associations could be partly explained by the fact that individuals who consume more nuts may also have healthier lifestyle habits. Fifth, although men with a history of known reproductive diseases were excluded, a standardized andrological physical examination (e.g., to diagnose subclinical varicoceles) and systematic hormonal assessment were not performed. Sixth, although associations with multiple sperm parameters were examined, no correction for multiple comparisons was applied, as all analyses were hypothesis‐driven and pre‐specified. Finally, it is essential to contextualize the study population. Our results are derived from a cohort of young, healthy, and mostly educated men from the general population. Therefore, these findings should not be directly extrapolated to infertile men or clinical fertility settings. Although the observed differences in sperm parameters were statistically significant, their clinical relevance within the normozoospermic range remains uncertain and requires confirmation in studies with fertility endpoints.

Despite these limitations, the present study also has notable strengths. This study is one of the few studies investigating the association between regular nut consumption and sperm quality parameters in healthy men from the general population. The analysis considered total mixed nuts (including almonds, pistachios, walnuts, and other nuts) consumption, enabling the evaluation of possible synergistic effects between their nutrients and bioactive compounds. Furthermore, semen analyses were conducted using a CASA system to enhance accuracy and reduce human error. Nut intake was categorized based on internationally recognized dietary recommendations to promote cardiovascular and metabolic health (≥ 30 g/day) [29], which facilitates the interpretation and application of the findings. Finally, the statistical models were adjusted for a wide range of relevant sociodemographic and lifestyle factors.

In conclusion, in this cross‐sectional study of young, healthy men from the general population, nut consumption was positively associated with several sperm quality parameters, although generalization beyond this group should be made with caution. Specifically, higher nut consumption was associated with greater total sperm count and sperm concentration. Moreover, men with higher nut consumption had 75% lower odds of abnormal total motility.

These findings add to the growing body of evidence linking specific food groups to male reproductive health. However, given the observational and cross‐sectional design of the study, causal inferences cannot be made. To translate these associations into clinical recommendations, longitudinal studies and controlled intervention trials are needed to establish causality, evaluate their impact on fertility outcomes, and elucidate the underlying biological mechanisms.

## Author Contributions

**Estefanía Dávila‐Córdova**: methodology, formal analysis, data curation, writing – original draft. **Nancy Babio**: conceptualization, methodology, supervision, project administration, funding acquisition, writing – original draft, writing – review and editing. **Cristina Valle‐Hita**: data curation, writing – review and editing. **María Fernández de la Puente**: investigation, data curation, writing – review and editing. **Alfonso Beltran‐Arasa**: data curation, writing – review and editing. **Miguel Cebrián‐Puig**: data curation, writing– review and editing. **Violeta Fambuena‐Perez**: data curation, writing – review and editing. **Iván García‐Serrano**: data curation, writing – review and editing. **Michelle M. Murphy**: data curation, writing – review and editing. **Jordi Salas‐Salvadó**: conceptualization, project administration, funding acquisition, supervision, writing – review and editing. **Albert Salas‐Huetos**: conceptualization, methodology, formal analysis, data curation, supervision, project administration, funding acquisition, writing – original draft, review and editing.

## Funding

The Led‐Fertyl study received support from several sources: the Spanish government's official funding agency for biomedical research, the Carlos III Health Institute (ISCIII), through the Fondo de Investigacion para la Salud (FIS); the European Union ERDF/ESF, “A way to make Europe”/“Investing in your future” (PI21/01447); and the Diputació de Tarragona (2021/11‐No.Exp. 8004330008‐2021‐0022642). Jordi Salas‐Salvadó is a distinguished senior researcher with support from the ICREA Academia Program. The ISCIII, the Spanish Ministry of Health, awarded Estefanía Dávila‐Córdova a Contrato Pre‐doctoral de Formación en Investigación en Salud (PFIS FI22/00018) of the Acción Estratégica en Salud program (AES).

## Conflicts of Interest

The authors declare no conflicts of interest.

## Supporting information




**Supplemental Figure 1**. Selection of participants for analysis.

**Supplemental Figure 2**. Associations (OR and its 95% confidence intervals) between categories of total nut consumption (servings per week) and abnormal sperm quality parameters (n = 222). Abbreviations: BMI, body mass index; METs, metabolic equivalents; spz, spermatozoa. Odds ratios were estimated using multivariable logistic regression models. Models were adjusted for age (years), smoking status (current, former, never), education (high school or less, college or high education), BMI (kg/m2), physical activity (METs min/week), sexual abstinence (days), energy intake (kcal/day) and dietary factors (consumption of vegetables, fruits, legumes, cereals, oils and fats, biscuits, dairy, meat, fish and beverages). * Indicate p < 0.05 between T1 (Reference) and T3.

**Supporting File 1**: andr70204‐sup‐0003‐Figure‐Captions.docx.

**Supplemental Table 1**. Multivariable‐adjusted *β*‐coefficients and 95% confidence intervals for the association between nut subtypes (almonds, pistachios, walnuts, and other nuts) modeled continuously per 1‐serving per day increase, and sperm quality parameters (n = 222).

**Supplemental Table 2**. Associations between nut consumption and sperm quality parameters, stratified by adherence to the Mediterranean diet (MEDAS).

## Data Availability

The data that support the findings of this study are available on request from the corresponding author. The data are not publicly available due to privacy or ethical restrictions.
