# Supplementary material for: Nut Consumption and Sperm Quality in Healthy Men: Results From the Led‐Fertyl Study
Source: Andrology. 2026 Feb 26;14(7):2262–73. doi: 10.1111/andr.70204 (PMC13592052; doi:10.1111/andr.70204)
Supplement: Supplementary file 1 [file ANDR-14-2262-s004.pdf]

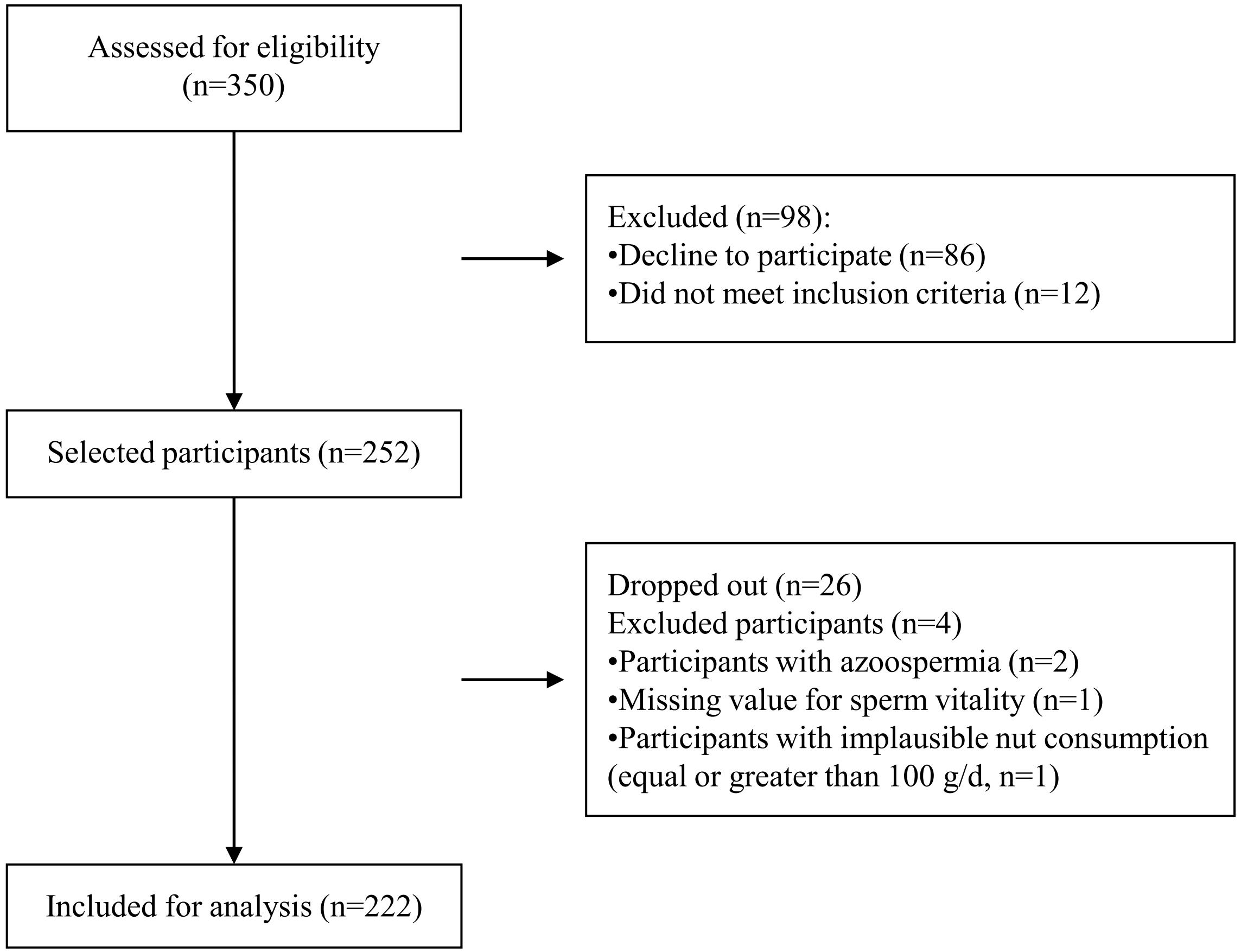
Assessed for eligibility
(n=350)
Excluded (n=98):
•Decline to participate (n=86)
•Did not meet inclusion criteria (n=12)
Selected participants (n=252)
Dropped out (n=26)
Excluded participants (n=4)
•Participants with azoospermia (n=2)
•Missing value for sperm vitality (n=1)
•Participants with implausible nut consumption (equal or greater than 100 g/d, n=1)
Included for analysis (n=222)
