# Supplementary material for: Nut Consumption and Sperm Quality in Healthy Men: Results From the Led‐Fertyl Study
Source: Andrology. 2026 Feb 26;14(7):2262–73. doi: 10.1111/andr.70204 (PMC13592052; doi:10.1111/andr.70204)
Supplement: Supplementary file 2 [file ANDR-14-2262-s005.pdf]

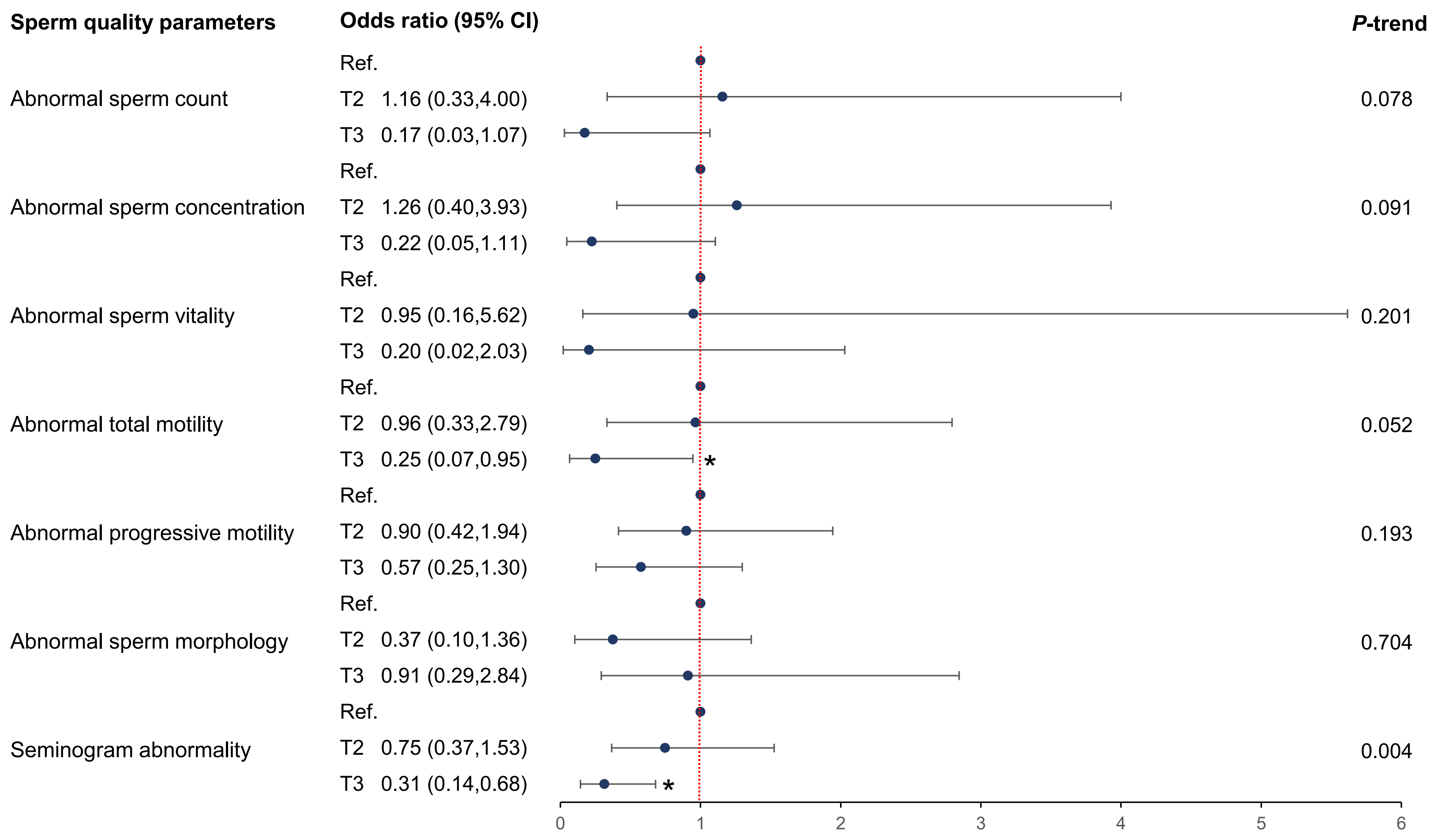
Sperm quality parameters
Odds ratio (95% CI)
P-trend
Abnormal sperm count
Ref.
T2 1.16 (0.33,4.00)
T3 0.17 (0.03,1.07)
0.078
Abnormal sperm concentration
Ref.
T2 1.26 (0.40,3.93)
T3 0.22 (0.05,1.11)
0.091
Abnormal sperm vitality
Ref.
T2 0.95 (0.16,5.62)
T3 0.20 (0.02,2.03)
0.201
Abnormal total motility
Ref.
T2 0.96 (0.33,2.79)
T3 0.25 (0.07,0.95)
*
0.052
Abnormal progressive motility
Ref.
T2 0.90 (0.42,1.94)
T3 0.57 (0.25,1.30)
0.193
Abnormal sperm morphology
Ref.
T2 0.37 (0.10,1.36)
T3 0.91 (0.29,2.84)
0.704
Seminogram abnormality
Ref.
T2 0.75 (0.37,1.53)
T3 0.31 (0.14,0.68)
*
0.004
0
1
2
3
4
5
6
