# Supplementary material for: Nut Consumption and Sperm Quality in Healthy Men: Results From the Led‐Fertyl Study
Source: Andrology. 2026 Feb 26;14(7):2262–73. doi: 10.1111/andr.70204 (PMC13592052; doi:10.1111/andr.70204)
Supplement: Supplementary file 3 [file ANDR-14-2262-s001.docx]

Supplemental Table 2. Associations between nut consumption and sperm quality parameters, stratified by adherence to the Mediterranean diet (MEDAS)

| Sperm quality parameters | | Categories of total nut consumption (servings per week) | | | | Continuous  (1-serving per day) |
| --- | --- | --- | --- | --- | --- | --- |
|  |  | <3 s/wk  (n=88) | ≥3 to <7 s/wk  (n=60) | ≥7 s/wk  (n=74) | P-trend |  |
| Total sperm count (x106 spz.)^a^ | Low MEDAS (n=64) | Ref. | -0.35 [-3.88, 3.19] | -0.88 [-5.44, 3.68] | 0.697 | -0.13 [-0.41, 0.16] |
|  | High MEDAS (n=158) | Ref. | 1.98 [-0.24, 4.21] | **3.67 [1.53, 5.82]** | **0.001** | **0.17 [0.06, 0.28]** |
| Sperm concentration (x106 spz./ml)^a^ | Low MEDAS (n=64) | Ref. | -0.63 [-2.63, 1.37] | -1.30 [-3.88, 1.28] | 0.293 | -0.12 [-0.27, 0.04] |
|  | High MEDAS (n=158) | Ref. | 1.27 [-0.01, 2.55] | **1.67 [0.44, 2.90]** | **0.008** | **0.08 [0.01, 0.14]** |
| Sperm vitality (%)^a^ | Low MEDAS (n=64) | Ref. | 0.06 [-0.43, 0.55] | 0.12 [-0.50, 0.74] | 0.684 | 0.01 [-0.03, 0.05] |
|  | High MEDAS (n=158) | Ref. | 0.20 [-0.09, 0.48] | 0.09 [-0.19, 0.36] | 0.537 | -0.01 [-0.02, 0.01] |
| Total motility (%) | Low MEDAS (n=64) | Ref. | 0.81 [-10.23, 11.86] | 2.31 [-11.93, 16.55] | 0.747 | 1.14 [-0.75, 1.02] |
|  | High MEDAS (n=158) | Ref. | 6.89 [-0.23, 14.01] | 5.00 [-1.86, 11.86] | 0.156 | 0.01 [-0.35, 0.36] |
| Progressive motility (%) | Low MEDAS (n=64) | Ref. | -0.61 [-12.00, 10.78] | -1.73 [-16.42, 12.95] | 0.814 | -0.18 [-1.09, 0.74] |
|  | High MEDAS (n=158) | Ref. | 6.50 [-0.93, 13.93] | 5.00 [-2.16, 12.16] | 0.173 | 0.03 [-0.33, 0.40] |
| Normal sperm morphology (%)^a^ | Low MEDAS (n=64) | Ref. | 0.25 [-0.44, 0.92] | 0.10 [-0.79, 0.99] | 0.669 | 0.02 [-0.04, 0.07] |
|  | High MEDAS (n=158) | Ref. | 0.27 [-0.23, 0.76] | 0.11 [-0.37, 0.59] | 0.651 | -0.01 [-0.03, 0.01] |

Multivariable linear regression models were fitted [β coefficient (95 % Confidence Interval)] adjusted for age (years), smoking status (current, former, never), education (high school or less, college or high education), BMI (kg/m2), physical activity (METs min/week), sexual abstinence (days), energy intake (kcal/day) and dietary factors (consumption of vegetables, fruits, legumes, cereals, oils and fats, biscuits, dairy, meat, ﬁsh, and beverages).

Abbreviations: BMI, body mass index; MEDAS, Mediterranean diet adherence screener; n, number of subjects; spz, spermatozoa.
