# Supplementary material for: Nut Consumption and Sperm Quality in Healthy Men: Results From the Led‐Fertyl Study
Source: Andrology. 2026 Feb 26;14(7):2262–73. doi: 10.1111/andr.70204 (PMC13592052; doi:10.1111/andr.70204)
Supplement: Supplementary file 4 [file ANDR-14-2262-s002.docx]

**Supplemental Table 1.** Multivariable-adjusted β-coefficients and 95% confidence intervals for the association between nut subtypes (almonds, pistachios, walnuts, and other nuts) modeled continuously per 1-serving per day increase, and sperm quality parameters (n=222).

| Sperm quality parameters | Almond consumption (s/day) | Pistachio consumption (s/day) | Walnut consumption (s/day) | Others nuts  consumption (s/day) |
| --- | --- | --- | --- | --- |
|  |  |  |  |  |
| Total sperm count (x10^6^ spz.) ^a^ | **4.36 (0.66, 8.07)** | **6.97 (1.83, 12.12)** | 3.00 (-0.31, 6.31) | **3.30 (0.48, 6.11)** |
| Sperm concentration (x10^6^ spz. /ml) ^a^ | 1.06 (-1.03, 3.15) | 1.98 (-0.92, 4.89) | 1.23 (-0.62, 3.08) | 1.38 (-0.20, 2.96) |
| Sperm vitality (%) ^a^ | -0.07 (-0.56, 0.43) | 0.32 (-0.37, 1.01) | -0.30 (-0.74, 0.14) | -0.10 (-0.47, 0.28) |
| Total motility (%) | 1.35 (-10.45, 13.15) | 13.52 (-2.82, 29.86) | -0.98 (-11.45, 9.50) | 1.90 (-7.06, 10.86) |
| Progressive motility (%) | -0.67 (-12.73, 11.38) | 12.43 (-4.28, 29.14) | -2.85 (-13.55, 7.84) | 1.27 (-7.88, 10.42) |
| Non-progressive motility (%) | 1.26 (-3.40, 5.91) | 0.89 (-5.60, 7.38) | 0.61 (-3.52, 4.75) | -0.19 (-3.73, 3.35) |
| Normal sperm morphology (%) ^a^ | 0.06 (-0.71, 0.83) | 0.23 (-0.84, 1.30) | -0.09 (-0.78, 0.59) | -0.02 (-0.60, 0.56) |

β-coefficients were estimated using multivariate linear regression models. Model was adjusted by age (years), smoking status (current, former, never), education (high school or less, college or high education), BMI (kg/m^2^), physical activity (METs min/week), sexual abstinence (days), energy intake (kcal/day) and dietary factors (consumption of vegetables, fruits, legumes, cereals, oils and fats, biscuits, dairy, meat, ﬁsh, and beverages). Bold values indicate p < 0.05.

^a^ Total sperm count, concentration, vitality and normal morphology were root-square transformed to approximate a normal distribution.

Abbreviations: BMI, body mass index; s, serving; spz, spermatozoa.
