# Supplementary material for: Nut Consumption and Sperm Quality in Healthy Men: Results From the Led‐Fertyl Study
Source: Andrology. 2026 Feb 26;14(7):2262–73. doi: 10.1111/andr.70204 (PMC13592052; doi:10.1111/andr.70204)
Supplement: Supplementary file 5 [file ANDR-14-2262-s003.docx]

**Supplemental Figure 1.** Selection of participants for analysis.

**Supplemental Figure 2.** Associations (OR and its 95% confidence intervals) between categories of total nut consumption (servings per week) and abnormal sperm quality parameters (n = 222).

Abbreviations: BMI, body mass index; METs, metabolic equivalents; spz, spermatozoa.

Odds ratios were estimated using multivariable logistic regression models. Models were adjusted for age (years), smoking status (current, former, never), education (high school or less, college or high education), BMI (kg/m2), physical activity (METs min/week), sexual abstinence (days), energy intake (kcal/day) and dietary factors (consumption of vegetables, fruits, legumes, cereals, oils and fats, biscuits, dairy, meat, ﬁsh and beverages). * Indicate p < 0.05 between T1 (Reference) and T3.
